# Heart rate fragmentation as a marker of autonomic function in healthy young individuals: insights from graded head-up tilt test and controlled breathing

**DOI:** 10.3389/fphys.2026.1880925

**Published:** 2026-07-03

**Authors:** Thaís Marques da Silva, Angelica Carandina, Costanza Scatà, Greta Salafia, Francesca Magna, Lucia Silvia Giobbi, Gabriel Dias Rodrigues, Eleonora Tobaldini, Luiz Eduardo Virgílio Silva, Helio Cesar Salgado, Rubens Fazan, Nicola Montano

**Affiliations:** 1 Department of Physiology, Ribeirao Preto Medical School, University of São Paulo, Ribeirao Preto, Brazil; 2 Department of Clinical Sciences and Community Health, Department of Excellence 2023-2027, Policlinico, Milan, Italy; 3 Department of Internal Medicine, Fondazione IRCCS Ca’ Granda Ospedale Maggiore Policlinico, Milan, Italy; 4 Department of Physiology and Pharmacology, Federal Fluminense University, Niterói, RJ, Brazil; 5 Department of Biomedical and Health Informatics, Children’s Hospital of Philadelphia, Philadelphia, PA, United States

**Keywords:** heart rate fragmentation, heart rate variability, parasympathetic modulation, head-up tilt, controlled breathing, symbolic analysis

## Abstract

**Introduction:**

Heart rate fragmentation (HRF) quantifies rapid beat-to-beat irregularity in cardiac intervals and has emerged as a predictor of adverse cardiovascular outcomes. While standard heart rate variability (HRV) indices primarily reflect autonomic modulation, HRF has been proposed to capture intrinsic sinoatrial node dynamics largely independent of autonomic influences. However, the extent to which HRF is shaped by cardiac autonomic balance in healthy individuals remains unclear. We examined whether HRF responds to controlled physiological shifts in autonomic modulation and investigated its relationship with established HRV indices.

**Methods:**

Twenty healthy adults (29 ± 4 years) underwent two complementary autonomic challenges: a graded head-up tilt test to progressively increase sympathetic drive and attenuate vagal cardiac modulation, and controlled breathing at 0.1 Hz to selectively enhance cardiac parasympathetic activity in a slow frequency band. HRF indices were quantified along with standard time- and frequency-domain HRV parameters and symbolic dynamic analysis. Associations between HRF and HRV indices were assessed using Spearman’s rank correlation coefficients.

**Results:**

Graded tilt produced a progressive reduction in HRF indices that closely paralleled decreases in vagally mediated HRV parameters. HRF indices, particularly the percentage of inflection points (PIP), showed strong correlations with the 2UV symbolic pattern (r = 0.74). Controlled breathing enhanced parasympathetic modulation and elicited a shift of respiratory oscillations from high- to low-frequency spectral bands. All variations corresponding to high frequencies (HF, 2UV%, PIP, and other indices related to more fragmented rhythms) decreased in favor of an increase in low-frequency variations (LF/HF, 0V%, 2LV%).

**Discussion:**

Rather than providing information purely about cardiovascular autonomic control, these findings indicate that, in healthy subjects, both HRV and HRF indices are strongly influenced by cardiorespiratory coupling. Collectively, these results provide the first evidence that HRF in healthy individuals is significantly influenced by autonomic activity, especially parasympathetic modulation. These findings challenge the prevailing view of HRF as a purely intrinsic, autonomic-independent marker, indicating that it is also influenced by the respiratory frequency range. Taken together, these observations indicate that HRF reflects autonomic mechanisms under physiological conditions. Whether this relationship is preserved or altered under pathological conditions remains to be established and warrants investigation in future studies.

## Introduction

Heart rate variability (HRV) describes fluctuations in cardiac intervals (CI) over time, providing valuable information about cardiovascular regulation (HRV Task Force, 1996; Shaffer et al., 2014). Cardiac autonomic modulation is the primary physiological substrate of HRV, and impairment of autonomic regulatory mechanisms generally reduces it. Accordingly, HRV indices are well-established biomarkers independently associated with increased cardiovascular risk across multiple conditions (Malliani et al., 1991; HRV Task Force, 1996; Shaffer et al., 2014).

Indices of HRV are derived from the series of CI fluctuations, with time- and frequency- domain (spectral) metrics being the most widely used (Malliani et al., 1991; Montano et al., 1994; HRV Task Force, 1996; Guzzetti et al., 2005). Spectral analysis decomposes CI variability into very-low-, low-, and high-frequency components. The high-frequency band, in particular, reflects vagal modulation of the heart, aligning with the respiratory cycle and capturing respiratory sinus arrhythmia (Malliani et al., 1991; Montano et al., 1994).

Beyond these established CI oscillations, ultrafast beat-to-beat variations termed Heart Rate Fragmentation (HRF) have been described more recently (Stein et al., 2008; Costa et al., 2017b, 2018). HRF represents a modern quantitative formulation of earlier observations of erratic and non-Gaussian sinus rhythm dynamics (Stein et al., 2008; Hayano and Yuda, 2019). These erratic fluctuations were reported to occur at frequencies beyond the range typically associated with parasympathetic activity, thus constituting a potentially independent biomarker of disorganized cardiac dynamics. The HRF literature provides consistent evidence linking elevated HRF indices to cardiovascular risk, aging, and renal function decline (Costa et al., 2017b, a, 2018). Our group has demonstrated that HRF is increased in experimental models of myocardial infarction at both early and late stages (4 and 12 weeks), even in the absence of significant changes in conventional HRV indices (Omoto et al., 2021). Moreover, we have reported elevated HRF in patients with type 2 diabetes mellitus (Aguiar Mesquita Galdino et al., 2023). Collectively, these findings highlight the potential of HRF as a sensitive and complementary tool for cardiovascular risk assessment, complementing traditional HRV analyses and enabling more refined stratification of autonomic and cardiac dysfunction.

Nevertheless, unlike several HRV indices whose physiological origins are well characterized, the mechanisms underlying HRF generation remain incompletely understood. Rather than autonomic regulation, the primary determinants of HRF are hypothesized to involve intrinsic sinoatrial mechanisms, including pacemaker cell dysfunction secondary to inflammation, fibrosis, degeneration, and calcification. From a mechanistic perspective, alterations in HRF may reflect disruptions in sinoatrial node dynamics, which can be interpreted within the coupled-clock model framework, where impaired interaction between membrane and calcium clocks leads to increased beat-to-beat irregularity (Lakatta et al., 2010; Yaniv et al., 2014, 2015).

This study aimed to elucidate the mechanisms underlying HRF generation by examining HRF behavior during controlled physiological shifts in autonomic balance and by characterizing its relationship with conventional HRV indices. We hypothesized that HRF is correlated with established HRV indices of autonomic modulation in healthy individuals. To test this hypothesis, we employed two well-established autonomic challenges that have been extensively used in the cardiovascular autonomic literature and whose physiological effects on autonomic regulation, as well as on conventional HRV indices, are well documented. Specifically, we used a graded head-up tilt test, a standardized maneuver known to progressively increase sympathetic activation and reduce vagal modulation, and controlled breathing at 0.1 Hz, a physiological intervention widely recognized for enhancing cardiac vagal modulation and inducing characteristic changes in HRV spectral indices through respiratory-baroreflex interactions. By combining these approaches, we sought to determine whether HRF varies consistently with shifts in autonomic tone and to clarify its physiological significance in healthy subjects.

## Methods

Healthy individuals were enrolled in the study between September and October 2025. Inclusion criteria comprised individuals of both sexes aged 18 years or older, with no chronic diseases and not receiving acute or chronic pharmacological treatment. Exclusion criteria included absence of stable sinus rhythm on electrocardiogram (e.g., pacemaker rhythm, atrial fibrillation, supraventricular or ventricular extrasystoles), history of surgery within the preceding 12 months, recent infectious illness (<3 months), participation in competitive sports, pregnancy or breastfeeding, and refusal to provide informed consent. The study was conducted in accordance with the Declaration of Helsinki, and the protocol was approved by the University of Milan Ethics Committee (approval code 88/25).

### Data collection protocols

For both protocols, the graded head-up tilt test and the controlled breathing protocol, participants were instructed to consume a light meal and to abstain from coffee and alcohol prior to each assessment. Single-channel ECG (lead I) signals were recorded at 1 kHz (PowerLab 4/40 and LabChart, ADInstruments), and subjects were instructed to remain still and silent during recordings. All sessions were conducted in the afternoon, between 2:00 PM and 6:00 PM, to minimize the influence of circadian variability in autonomic nervous system activity.

#### Graded head-up tilt test

Following a 5-minute adaptation period in the supine position, participants underwent a 10-minutes ECG recording at rest (baseline), followed by a 10-minute graded head-up tilt test at a randomly assigned table angle (15°, 45°, or 75°), using a motorized tilt table. After each tilt phase, subjects were returned to the supine position for a 5-minute recovery period before being exposed to the next randomly assigned angle (Montano et al., 1994). Blood pressure was measured at baseline and every 3 minutes during head-up tilt using an automated oscillometric cuff device (Edan iM3 Vital Signs Monitor, China). Respiratory activity was continuously monitored during the graded head-up tilt protocol using a thoracic belt to allow assessment of respiratory frequency across all experimental conditions. Tilt angles of 15°, 45°, and 75° were selected to provide distinct levels of orthostatic stress and autonomic engagement, following the graded tilt protocol of Montano et al. (1994), which demonstrated progressive sympathovagal changes across these inclinations.

#### Controlled breathing

In the week following the tilt test, the controlled breathing protocol was carried out in the same volunteers. After a 5-minute adaptation period in the supine position, subjects underwent a 5-minutes ECG recording during spontaneous breathing (baseline), followed by 5 minutes of slow-paced breathing at 6 breaths per minute (0.1 Hz; resonance breathing) (Nolan et al., 2005; Brown et al., 2013), and a subsequent 5-minutes spontaneous-breathing recovery period.

The 0.1 Hz breathing protocol was selected because it represents a well-established autonomic challenge that has been extensively used to investigate cardiovascular autonomic regulation and heart rate variability. Breathing at this frequency is known to elicit characteristic and reproducible changes in conventional HRV indices, reflecting enhanced cardiac vagal modulation and increased coupling among respiratory, cardiovascular, and baroreflex oscillations (Sevoz-Couche and Laborde, 2022; Baselli et al., 1994). In particular, 0.1 Hz breathing promotes temporal coherence between respiratory, blood pressure, and heart rate fluctuations, maximizing respiratory-mediated vagal influences on the sinoatrial node and producing marked changes in the spectral characteristics of HRV. The use of this well-characterized physiological maneuver therefore provided a suitable framework for evaluating whether HRF indices respond consistently to a condition associated with augmented parasympathetic cardiovascular modulation.

Adherence to the imposed respiratory rhythm was verified using a thoracic belt. Pacing was provided by the Breathe mobile application (Joachim Noh, iOS), which delivered synchronized visual and auditory guidance.

### Data analysis

ECG recordings were processed using LabChart software to detect QRS complexes and generate successive RR interval (RRi) series. Artifacts and ectopic beats were identified using a median-based adaptive window and corrected by linear interpolation using PyBioS software (Silva et al., 2020). The proportion of corrected RRi never exceeded 3% of the total recording. HRF and standard HRV analyses were subsequently performed on the corrected RRi series using the same software.

### Analysis of heart rate fragmentation

For HRF analysis, all RRi series were transformed into ternary symbolic sequences based on first-order differences between successive RRi. Each beat was assigned the symbol “+1”, “0”, or “-1” when the difference relative to the preceding interval was positive, zero, or negative, respectively. The percentage of inflection points (PIP) was then calculated from the ternary symbolic series. Next, sequences of four consecutive symbols (“words”) were classified according to their number of inflection points: W_0_, W_1_, W_2_, and W_3_ represent patterns containing zero, one, two, and three inflection points, respectively. Words were further categorized according to the type of inflection: transitions between “-1” and “1” were labeled as *hard* (W^H^), transitions between “-1” (or “1”) and “0” as *soft* (W^S^), and *mixed* (W^M^) sequences contained both types. A threshold of 5 ms was applied as the minimum difference required for successive RRi values to be considered distinct. In this study, a 5 ms threshold was adopted, consistent with prior work employing thresholds of 4 or 5 ms range, which aligns with the intrinsic temporal resolution of RR interval measurements derived from standard clinical ECG recordings sampled at 200–250 Hz (Costa et al., 2017b, a, 2018).

### Standard measures of heart rate variability

Standard HRV analysis included time- and frequency- domain measures: the standard deviation of normal-to-normal intervals (SDNN), the root mean square of successive differences (RMSSD), and spectral power in the low-frequency (LF: 0.04–0.15 Hz) and high-frequency (HF: 0.15–0.40 Hz) bands. The LF/HF ratio was computed as an index of sympathovagal balance (Malliani et al., 1991; HRV Task Force, 1996). Spectral analysis was performed on stationary RRi series comprising approximately 400 consecutive RRi for each condition.

Nonlinear HRV analysis was additionally performed using symbolic dynamics (Porta et al., 2001, 2007a). In this approach, the RRi series was partitioned into six equal-amplitude levels, and each interval was assigned a symbol from 0 to 5 according to its level. Sequences of three consecutive symbols were classified into three pattern families based on their directional variation: zero variation (0V), in which all three symbols are identical; two like variations (2LV), in which consecutive symbol changes occur in the same direction; and two unlike variations (2UV), in which changes alternate direction, forming a local peak or trough. The relative frequency of each pattern (0V%, 2LV%, and 2UV%) was computed. Prior work has established that 0V% reflects sympathetic modulation, whereas 2LV% and 2UV% predominantly mirror parasympathetic influences (Porta et al., 2007a; Silva et al., 2017).

### Statistical analysis

Data normality was assessed using the Shapiro–Wilk test. For the graded head-up tilt protocol, differences across tilt angles were analyzed using a nonparametric one-way repeated-measures analysis (Friedman test) followed by Dunn’s *post hoc* correction. In the controlled breathing protocol, differences between baseline and slow-paced breathing were evaluated using the Wilcoxon signed-rank test. Associations between HRF and HRV indices were examined using Spearman’s rank correlation coefficients in both protocols. The influence of respiratory frequency on PIP was evaluated using regression analysis. Correlation analyses were performed on data pooled across all conditions within each protocol; complementary analyses restricted to the individual conditions showed associations of similar direction and statistical significance. To control for multiple comparisons, p-values from the correlation analyses were adjusted using the Benjamini–Hochberg false discovery rate (FDR) correction. Statistical significance was set at P<0.05. Data are presented as median [Q1-Q3], except for demographic characteristics and respiratory frequency which are presented as mean ± SD.

## Results

### Graded head-up tilt test

A total of 20 healthy participants (9 men and 11 women) with a mean age of 29 ± 4 years were enrolled. The mean body mass index of the cohort was 24 ± 4 kg/m².

All participants completed the protocol without adverse events. Mean arterial pressure was modestly higher at 75° and 45°compared with baseline and 15°. Heart rate showed a significant progressive increase from baseline at 45° and 75° angles (**Table 1**).

**Table 1:** Linear and nonlinear heart rate variability indices under baseline and head-up tilt at different angles (15°, 45°, and 75°).

Indices	Baseline	15°	45°	75°
MAP	86.0 [78.3, 94.5]	86.5 [79.0, 93.0]	92 [84.3, 98.5]^ab^	89.0 [85.3, 100.0]^ab^
HR (bpm)	69.2 [61.8, 79.0]	69.0 [60.7, 76.4]	75.9 [70.2, 89.2]^ab^	83.5 [75.2, 91.3]^abc^
SDNN (ms)	47.5 [40.6, 51.7]	54.0 [37.9, 65.7]	49.2 [38.2, 61.1]	45.7 [32.4, 62.1]
RMSSD (ms)	35.4 [22.0, 42.7]	32.5 [27.9, 46.9]	22.7 [18.7, 30.8]^b^	19.2 [14.9, 26.9]^ab^
HF (nu)	35.2 [27.8, 49.7]	35.7 [19.9, 53.1]	21.2 [10.2, 32.8]^ab^	19.9 [10.2, 23.0]^ab^
LF/HF	1.8 [1.0, 2.6]	1.8 [0.9, 4.1]	3.7 [2.1, 8.8]^ab^	4.0 [3.4, 8.8]^ab^
0V (%)	19.7 [13.1, 41.6]	24.9 [12.8, 39.8]	37.7 [30.7, 54.6]^ab^	44.2 [35.2, 52.1]^ab^
2LV (%)	10.5 [5.8, 16.1]	10.2 [5.8, 16.5]	6.6 [3.3, 8.1]^ab^	6.0 [3.6, 10.0]^ab^
2UV (%)	12.6 [7.6, 20.4]	11.8 [7.4, 17.0]	5.6 [2.9, 9.0]^ab^	3.8 [2.8, 7.2]^ab^

Median [Q1, Q3]. ^a, b, c^P<0.05, a vs. basal, b vs. 15°, c vs. 45°. MAP, Mean Arterial Pressure; HR, Heart rate; nu, normalized units.

As expected, and consistent with previous reports (Montano et al., 1994; Porta et al., 2007b), both linear and nonlinear HRV indices confirmed that the graded head-up tilt test effectively shifted cardiac autonomic balance toward sympathetic predominance. RMSSD, HFnu 2LV%, and 2UV% decreased progressively, while LF/HF and 0V% increased with increasing tilt angle (**Table 1**).

**Figure 1:**
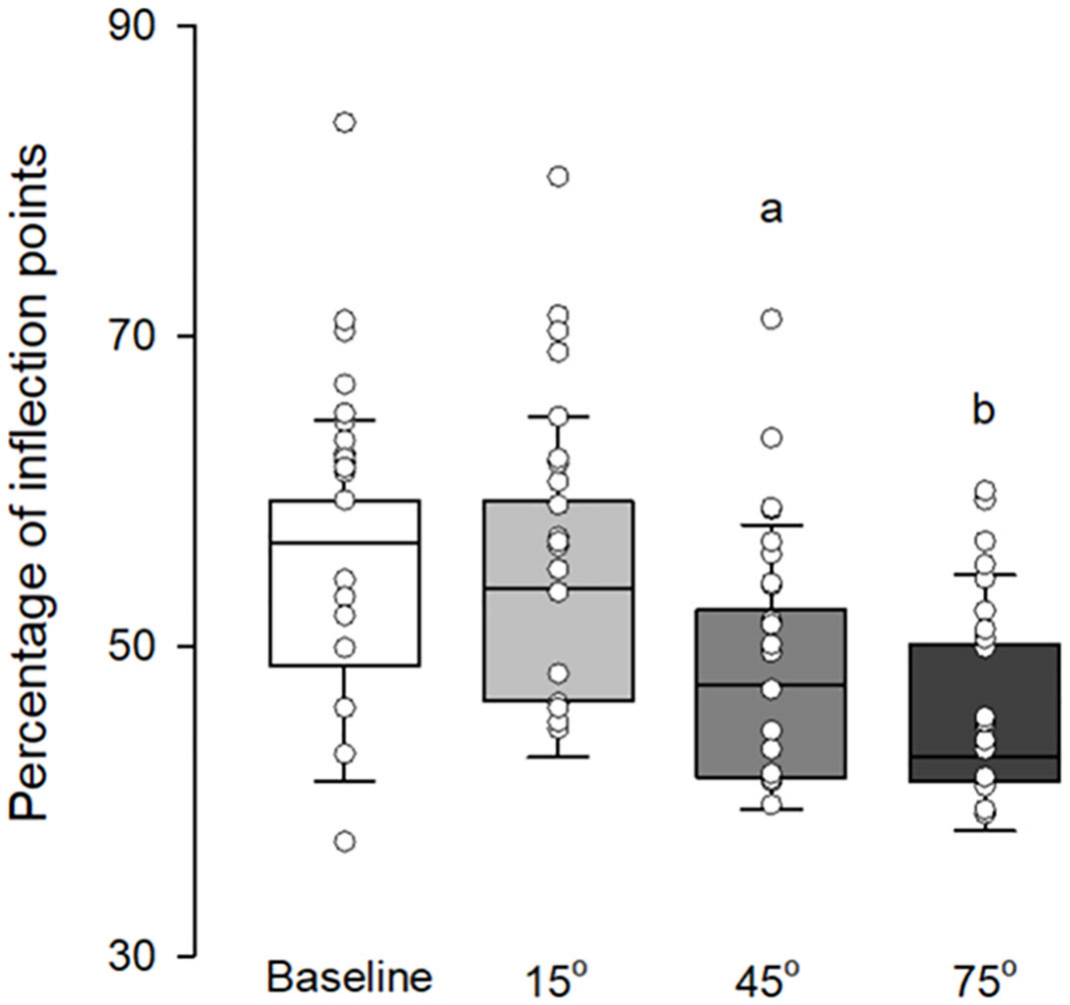
Percentage of inflection points (PIP) at baseline and during graded head-up tilt (15°, 45°, and 75°). Data are presented as median and interquartile range with individual data points. Statistical comparisons were performed using one-way repeated-measures analysis (Friedman test) followed by Dunn’s *post hoc* test. *p* < 0.05 vs. baseline (a); *p* < 0.05 vs. baseline and 15° (b).

**Figure 2:**
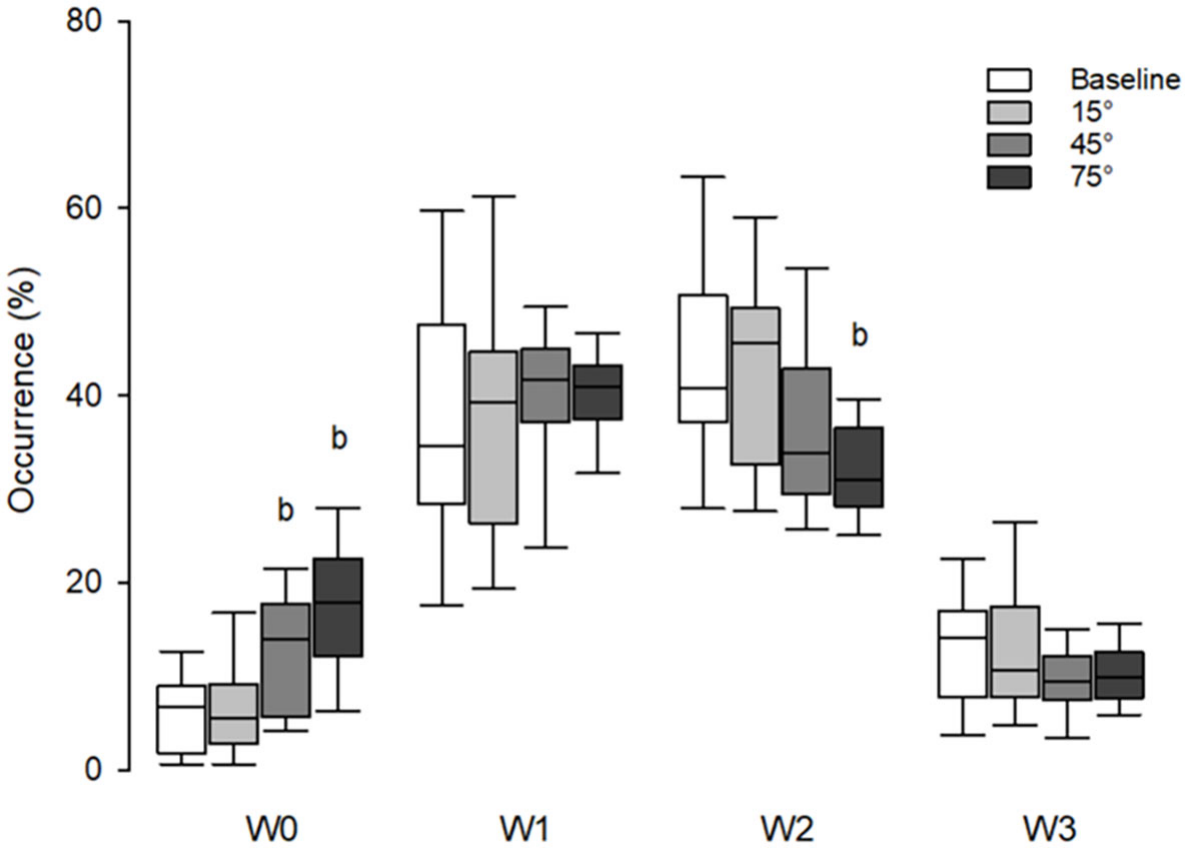
Occurrence (%) of symbolic patterns with zero (W_0_), one (W_1_), two (W_2_), and three (W_3_) inflection points at baseline and during graded head-up tilt (15°, 45°, and 75°). Data are presented as median and interquartile range. Statistical comparisons were performed using one-way repeated-measures analysis (Friedman test) followed by Dunn’s *post hoc* test. *p* < 0.05 vs. Baseline and 15° (b).

**Figure 3:**
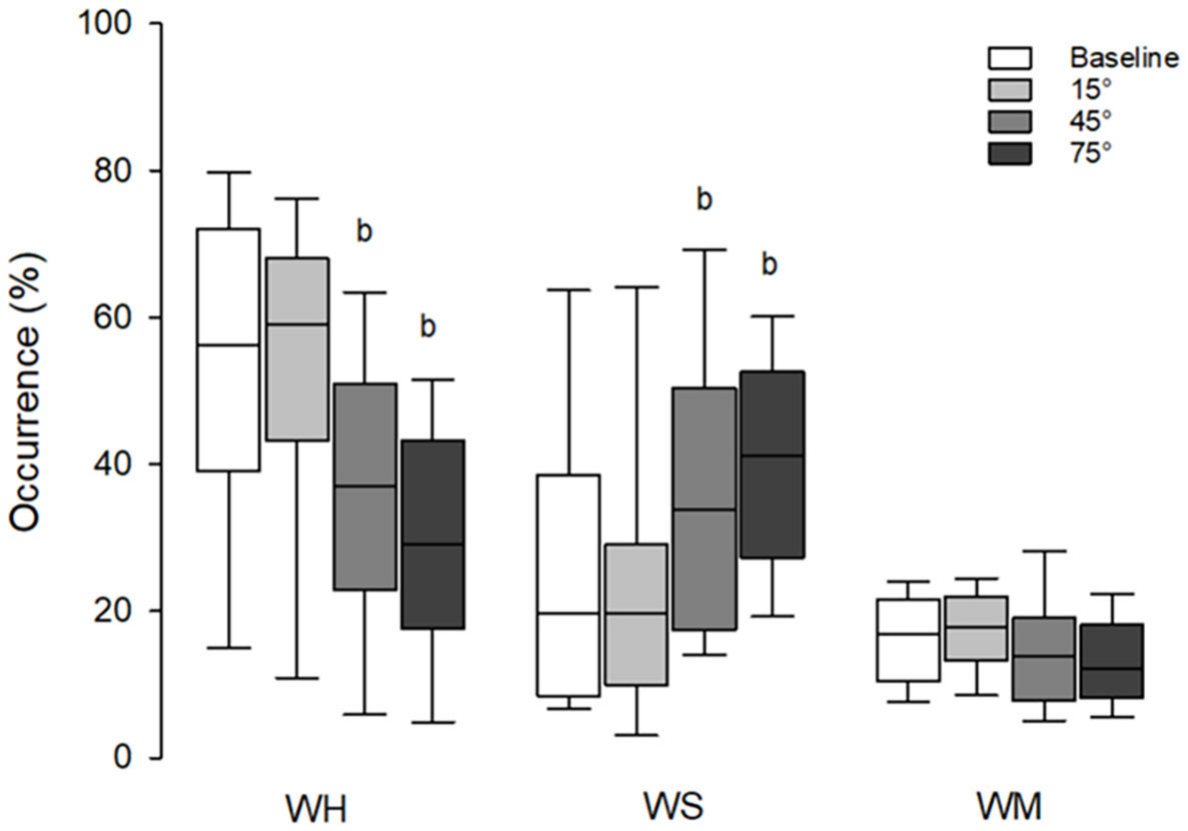
Occurrence (%) of symbolic patterns with hard (W^H^), soft (W^S^), and mixed (W^M^) inflection points at baseline and during graded head-up tilt (15°, 45°, and 75°). Data are presented as median and interquartile range. Statistical comparisons were performed using one-way repeated-measures analysis (Friedman test) followed by Dunn’s *post hoc* test. *p* < 0.05 vs. Baseline and 15° (b).

**Figures 1**–**3** depict HRF patterns across the different tilt angles. Indices associated with high fragmentation (PIP, W_2_, and W^H^) decreased progressively with increasing tilt angle, reaching their lowest values at 75° relative to baseline. Conversely, indices reflecting less fragmented patterns, such as W_0_ and W^S^, increased progressively, with their highest values observed at 75°. All HRV and HRF indices returned to baseline values during the recovery phase at all evaluated angles, confirming that the preceding tilt did not influence subsequent measurements.

Respiratory frequency was analyzed across tilt conditions using a one-way repeated-measures ANOVA and remained stable throughout the protocol, with mean values of 15 ± 4 breaths/min at baseline, 15 ± 3 breaths/min at 15°, 14 ± 4 breaths/min at 45°, and 14 ± 3 breaths/min at 75°, with no significant differences between conditions (p = 0.159). Although respiratory rate did not differ across tilt angles, respiratory frequency emerged as a significant predictor of PIP in the regression analysis (p < 0.001).

Correlation analyses revealed that PIP was positively associated with 2UV%, but not 2LV%, both symbolic patterns linked to parasympathetic cardiac modulation. Conversely, PIP showed negative correlations with the LF/HF ratio, an index of sympathovagal balance, and with 0V%, a marker associated with sympathetic modulation (**Table 2**).

**Table 2:** Correlation analysis between heart rate fragmentation indices and heart rate variability parameters during the graded head-up tilt test.

Indices	SDNN (ms)	RMSSD (ms)	LF/HF	0V (%)	2LV (%)	2UV (%)
PIP	-0.17	0.21	**-0.39**	**-0.45**	0.09	**0.74**
	0.175	0.086	**0.001**	**<0.001**	0.523	**<0.001**
W_0_	-0.02	**-0.51**	**0.76**	**0.77**	**-0.47**	**-0.89**
	0.886	**<0.001**	**<0.001**	**<0.001**	**<0.001**	**<0.001**
W_1_	**0.29**	0.09	-0.07	0.07	0.21	**-0.36**
	**0.014**	0.526	0.618	0.611	0.088	**0.002**
W_2_	-0.06	0.26	**-0.43**	**-0.45**	0.18	**0.70**
	0.634	0.293	**<0.001**	**<0.001**	0.159	**<0.001**
W_3_	**-0.35**	-0.19	0.09	0.03	**-0.32**	**0.29**
	**0.003**	0.135	0.511	0.841	**0.008**	**0.014**
W^H^	**0.54**	**0.91**	**-0.76**	**-0.90**	**0.68**	**0.88**
	**<0.001**	**<0.001**	**<0.001**	**<0.001**	**<0.001**	**<0.001**
W^S^	**-0.52**	**-0.86**	**0.68**	**0.86**	**-0.61**	**-0.88**
	**<0.001**	**<0.001**	**<0.001**	**<0.001**	**<0.001**	**<0.001**
W^M^	-0.05	0.11	-0.19	**-0.30**	0.06	**0.57**
	0.659	0.446	0.139	**0.012**	0.634	**<0.001**

Values represent Spearman’s correlation coefficients (upper value) and corresponding p values (lower value). Bold values indicate statistically significant correlations (P < 0.05).

Further notable associations were identified between HRF subindices and conventional HRV measures. W^H^ showed strong positive correlations with RMSSD and 2UV%, and a strong negative correlation with 0V%, consistent with a parasympathetically driven profile. Conversely, W^S^ displayed the opposite pattern, strong negative correlations with RMSSD and 2UV%, and a positive correlation with 0V%, indicating a close association with sympathetic cardiac modulation (**Table 2**).

**Figure 4:**
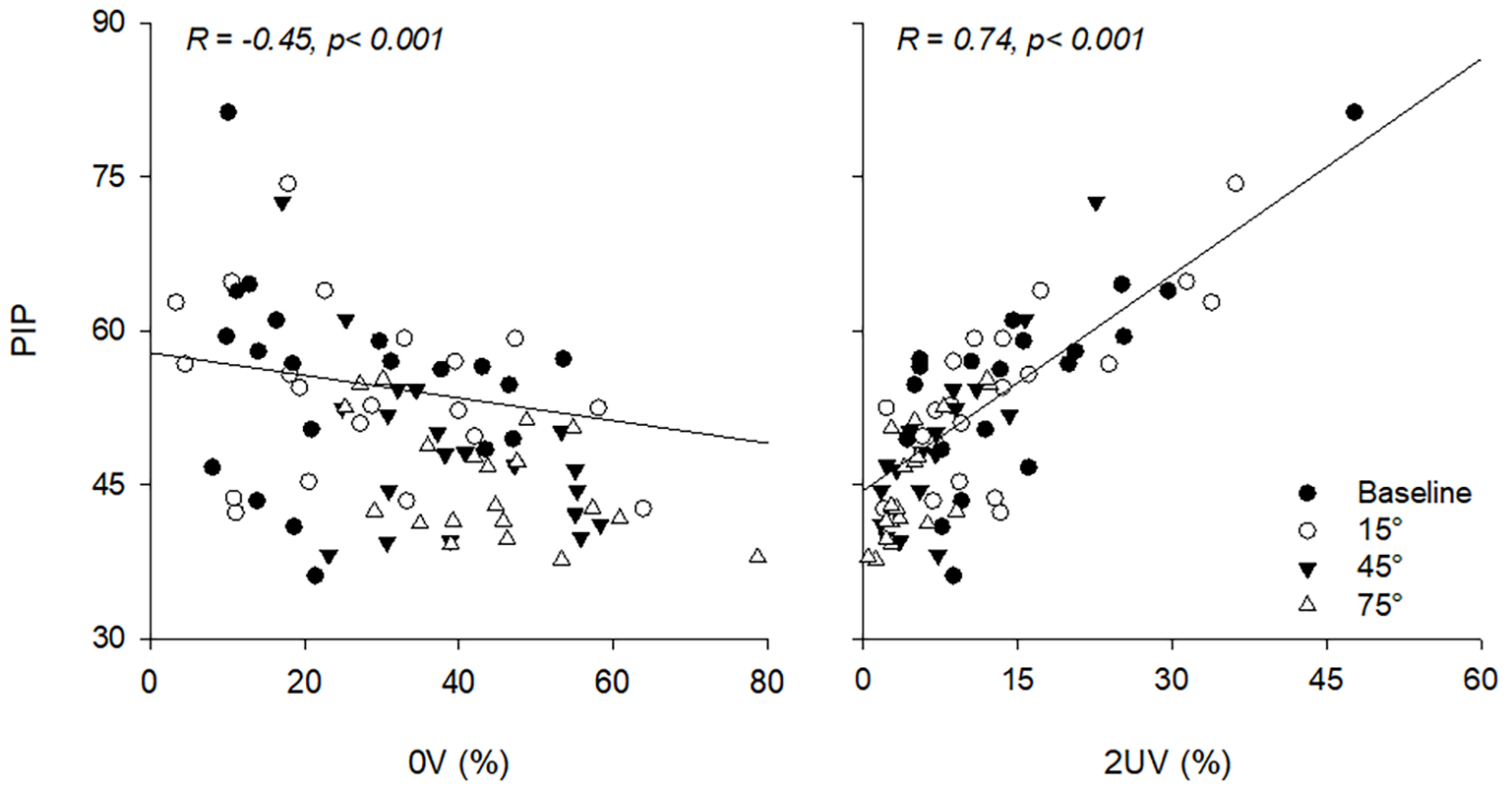
Scatter plots illustrating the relationship between the percentage of inflection points (PIP) and 0V% (left panel) and 2UV% (right panel) across all experimental conditions. Each symbol represents an individual observation. Spearman’s correlation coefficient (r) is indicated in each panel.

**Figure 4** illustrates the association between PIP and the two symbolic dynamics indices showing the strongest overall correlations: 0V% and 2UV%.

### Controlled breathing

A total of 18 healthy participants (9 men and 9 women) with a mean age of 29 ± 4 years were included. Two of the 20 eligible subjects could not complete assessment due to technical issues. All 18 included participants successfully completed the protocol without adverse events.

HRV analysis revealed increases in the time- domain indices SDNN and RMSSD, reflecting enhanced overall HRV and increased parasympathetic modulation. Spectral analysis showed a rise in the LF/HF ratio and a reduction in HFnu, while symbolic dynamics analysis demonstrated increases in 0V% and 2LV% and a decrease in 2UV% (**Table 3**).

**Table 3:** Linear and nonlinear heart rate variability indices under spontaneous and controlled breathing.

Indices	Spontaneous breathing	Controlled breathing
Heart rate (bpm)	64.8 [55.3, 71.6]	67.8 [57.6, 73.3]
SDNN (ms)	49.7 [40.8, 59.4]	81.3 [59.0, 105.3]*
RMSSD (ms)	34.4 [23.3, 52.7]	43.8 [29.4, 75.0]*
HF (nu)	44.6 [27.1, 61.1]	8.0 [4.0, 15.3]*
LF/HF	1.2 [0.6, 2.7]	11.6 [5.5, 24.1]*
0V (%)	18.3 [11.3, 30.2]	25.9 [18.4, 28.3]*
2LV (%)	10.9 [8.2, 19.3]	20.4 [17.0, 26.5]*
2UV (%)	13.0 [7.8, 22.2]	4.3 [3.3, 7.0]*

Median [first, third quartiles]. *P<0.05 vs. spontaneous breathing.

**Figure 5:**
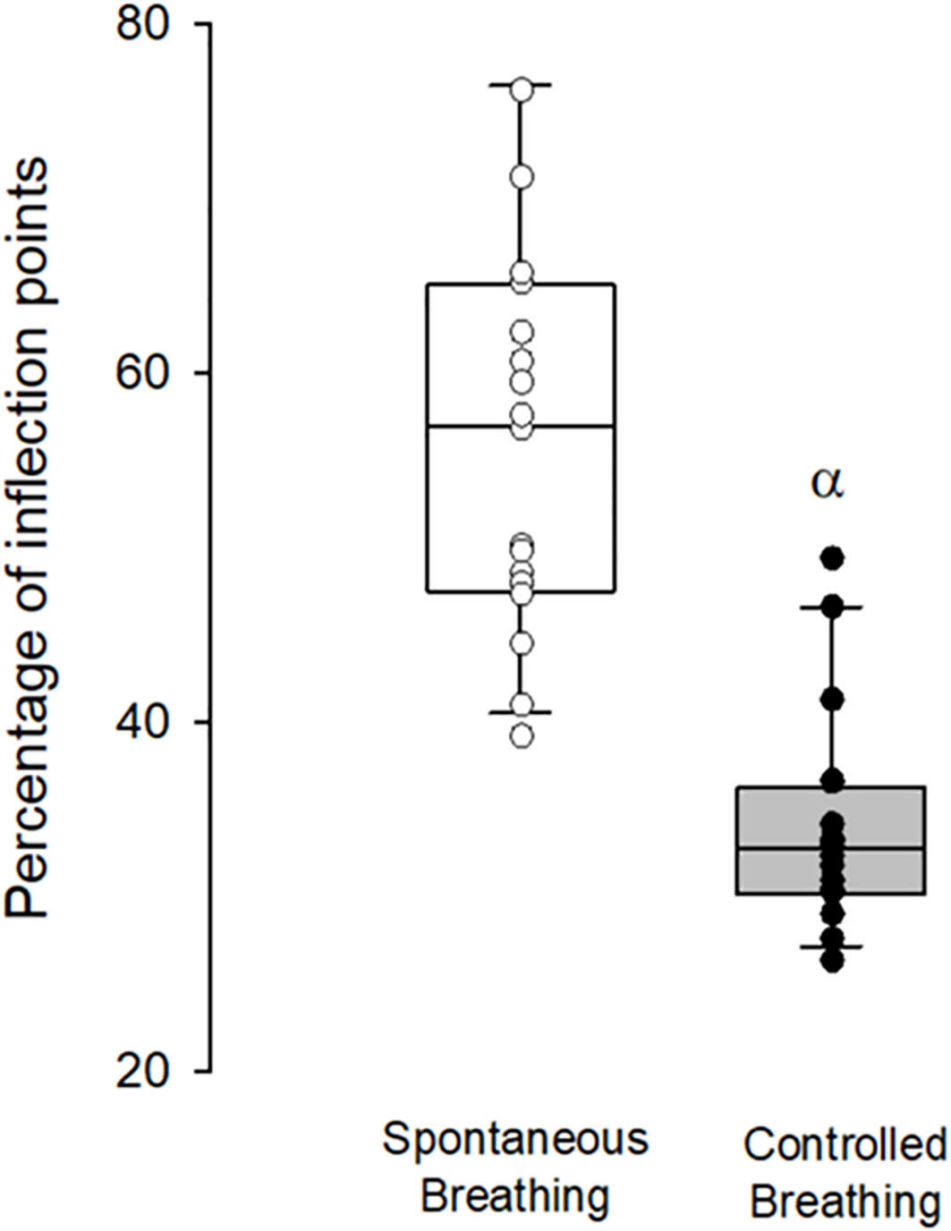
Percentage of inflection points (PIP) during spontaneous and controlled breathing. Data are presented as median and interquartile range with individual data points. Statistical comparison was performed using a Wilcoxon signed-rank test. *p* < 0.05 vs. spontaneous breathing (α).

**Figure 6:**
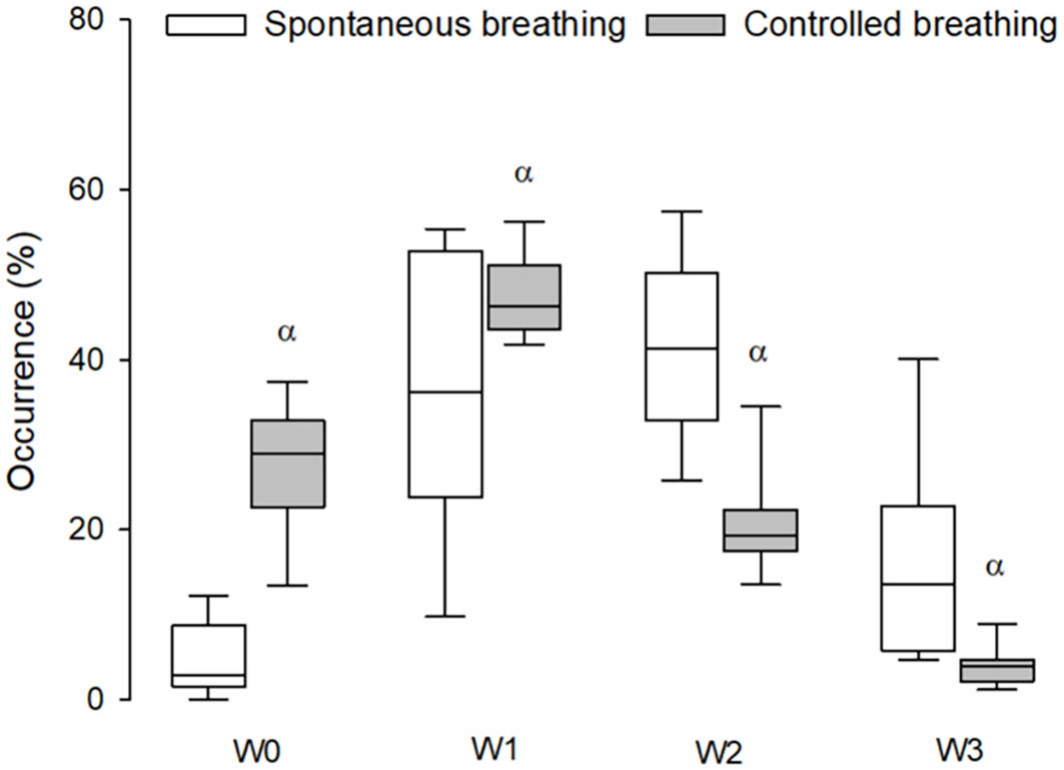
Occurrence (%) of symbolic patterns with zero (W_0_), one (W_1_), two (W_2_), and three (W_3_) inflection points during spontaneous and controlled breathing. Data are presented as median and interquartile range. Statistical comparisons were performed using a Wilcoxon signed-rank test. *p* < 0.05 vs. spontaneous breathing (α).

**Figure 7:**
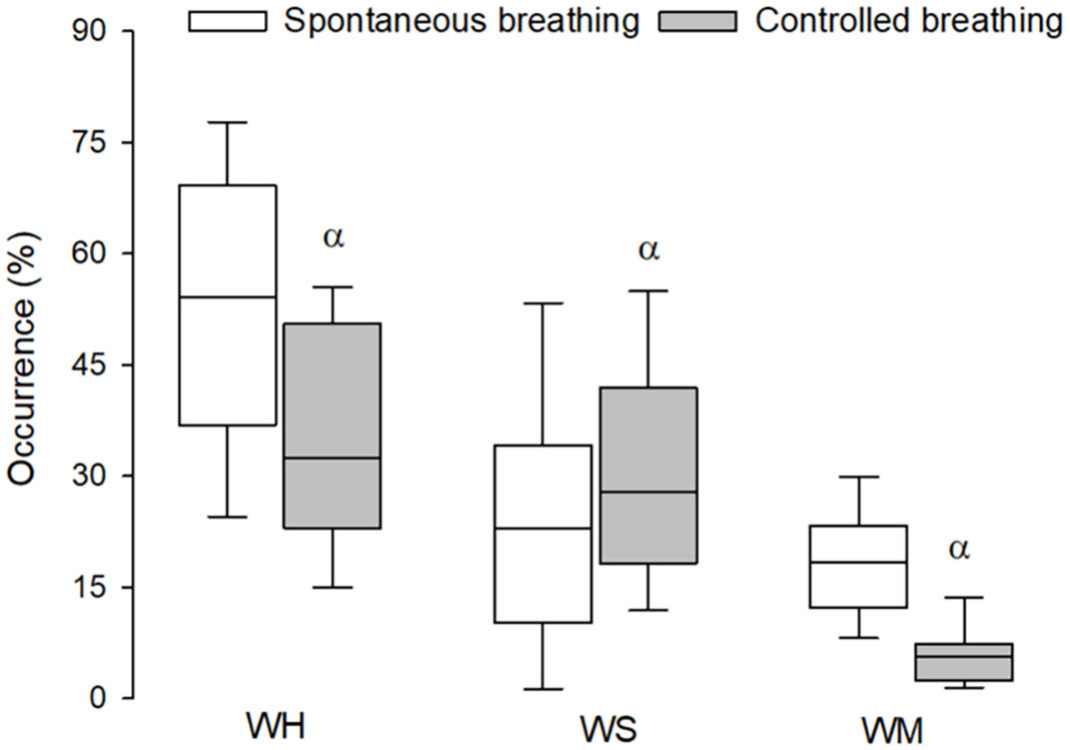
Occurrence (%) of symbolic patterns with hard (W^H^), soft (W^S^), and mixed (W^M^) inflection points during spontaneous and controlled breathing. Data are presented as median and interquartile range. Statistical comparisons were performed using a Wilcoxon signed-rank test. *p* < 0.05 vs. spontaneous breathing (α).

**Figures 5**–**7** illustrate HRF changes in response to controlled breathing. Relative to spontaneous breathing, controlled breathing was associated with a reduction in the most fragmented indices (PIP, W_2_, W_3_, W^H^, and W^M^) and a concomitant increase in the less fragmented indices (W_0_, W_1_, and W^S^).

Correlation analysis identified a positive association between PIP and 2UV%. Conversely, PIP was negatively correlated with both the LF/HF ratio and 2LV%. No significant correlation was found between PIP and 0V%.

**Figure 8:**
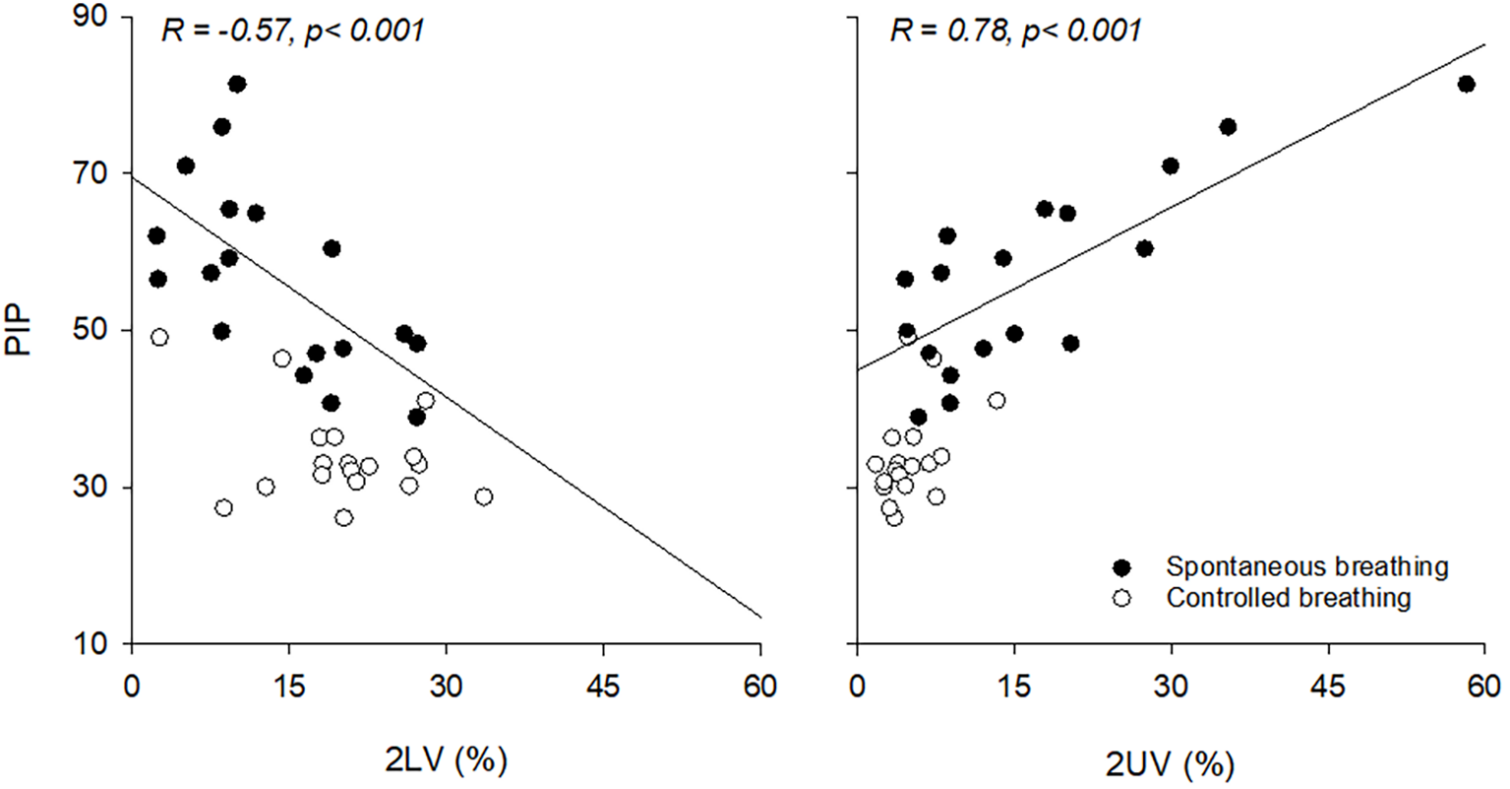
Scatter plots illustrating the relationship between the percentage of inflection points (PIP) and 2LV% (left panel) and 2UV% (right panel) during spontaneous and controlled breathing. Each symbol represents an individual observation. Spearman’s correlation coefficient (r) is indicated in each panel.

Among the remaining HRF subindices, W_0_ exhibited a strong positive association with the LF/HF ratio and a strong negative association with 2UV%. **Figure 8** illustrates the scatter plots for the correlations between PIP and the 2LV% and 2UV% symbolic dynamics indices. All correlation coefficients are reported in **Table 4**.

**Table 4:** Correlation analysis between heart rate fragmentation indices and heart rate variability parameters during spontaneous and controlled breathing.

Indices	SDNN (ms)	RMSSD (ms)	0V (%)	2LV (%)	2UV (%)
PIP	**-0.62**	-0.25	-0.16	-**0.57**	**0.78**
	**<0.001**	0.178	0.399	**<0.001**	**<0.001**
W_0_	**0.51**	0.10	0.33	**0.43**	**-0.85**
	**0.003**	0.586	0.069	**0.017**	**<0.001**
W_1_	**0.52**	**0.41**	-0.17	**0.72**	-0.19
	**0.002**	**0.020**	0.366	**<0.001**	0.332
W_2_	**-0.64**	-0.29	-0.14	**-0.56**	**0.73**
	**<0.001**	0.116	0.426	**<0.001**	**<0.001**
W_3_	**-0.72**	**-0.41**	-0.04	**-0.66**	**0.64**
	**<0.001**	**0.020**	0.811	**<0.001**	**<0.001**
W^H^	0.27	**0.64**	**-0.75**	0.15	**0.75**
	0.149	**<0.001**	**<0.001**	0.426	**<0.001**
W^S^	**-0.42**	**-0.73**	**0.75**	-0.19	**-0.63**
	**0.020**	**<0.001**	**<0.001**	0.324	**<0.001**
W^M^	**-0.64**	-0.34	-0.08	**-0.60**	**0.73**
	**<0.001**	0.0633	0.649	**<0.001**	**<0.001**

Values represent Spearman’s correlation coefficients (upper value) and corresponding p values (lower value). Bold values indicate statistically significant correlations (P < 0.05).

## Discussion

The present study examined, for the first time, HRF index behavior in response to two physiological maneuvers known to modulate cardiac autonomic control. Our main results are: i) indices associated with high fragmentation progressively decreased during the graded head-up tilt test, closely paralleling reductions in vagally mediated HRV parameters; ii) with regard to the changes observed during controlled breathing at 0.1 Hz, a reduction in all ultra-high-frequency components of the HRF was found; iii) PIP and 2UV% indices exhibited a strong degree of correlation across both autonomic challenges. During graded head-up tilt, sympathetic outflow progressively increases while vagal cardiac modulation is attenuated. Conventional HRV indices responded in accordance with this autonomic shift, consistent with findings from prior experiments in our laboratory (Montano et al., 1994). Respiratory monitoring is an important methodological consideration in HRV studies, as respiratory frequency, tidal volume, and breathing pattern can substantially influence respiratory sinus arrhythmia and, consequently, the interpretation of autonomic indices (Hirsch and Bishop, 1981; Eckberg, 2003). In the present study, although respiratory frequency was significantly associated with PIP in the regression analysis, respiratory rate remained stable across tilt conditions. This suggests that the observed association likely reflects interindividual variability in spontaneous breathing rate rather than a systematic respiratory effect induced by graded tilt.

Controlled breathing, by contrast, enhanced vagal cardiac influence, given that respiratory sinus arrhythmia is the principal parasympathetic determinant of HRV. Accordingly, classical HRV indices reflected an overall increase in HRV, accompanied by a spectral shift of respiratory oscillations from the high-frequency band into the low-frequency range (0.1 Hz, matching the imposed breathing rate).

This study provides the first demonstration that HRF indices change during maneuvers that shift cardiac autonomic balance in healthy individuals. The reduction in HRF during a procedure known to alter autonomic modulation is, at first glance, somewhat unexpected. Earlier studies proposed that fragmented heart rate patterns arise from mechanisms independent of autonomic influence, reflecting ultra-fast fluctuations occurring at frequencies above the typical high-frequency band associated with parasympathetic activity (Stein et al., 2008; Costa et al., 2017b, a). Those interpretations were derived primarily from studies involving older adults and patients with cardiovascular disease (Costa et al., 2018, 2021b, a; Costa and Goldberger, 2019). The present findings extend this framework to healthy individuals under physiological conditions and suggest that, in the absence of pathological alterations, HRF indices are not exclusively linked to erratic sinus rhythms operating independently of autonomic control. Rather, HRF may be influenced by the same respiratory frequency range, making HRF indices sensitive to changes in vagal modulation. We therefore propose that the progressive reduction in HRF observed during graded tilt reflects a decrease in the vagally mediated component of heart rate dynamics, rather than alterations in intrinsic sinoatrial activity.

Consistent with this interpretation, significant correlations between HRF and vagally associated HRV indices were observed during graded tilt, reinforcing the notion that HRF indices track shifts in parasympathetic activity. The strongest association was found between PIP and the 2UV% pattern from symbolic dynamics (r = 0.74). Despite relying on distinct computational frameworks, both indices quantify rapid beat-to-beat variability considered to reflect vagally mediated oscillatory activity, suggesting that HRF and symbolic dynamics capture overlapping physiological information in healthy subjects. Findings for W^H^ mirrored those for PIP, further supporting this interpretation.

A further consideration concerns the spectral proximity between the high-frequency band of respiratory sinus arrhythmia and the putative ultra-high-frequency band of HRF. Whether genuinely ultra-fast oscillations occur in healthy individuals remains uncertain, as the intrinsic cardiac alterations hypothesized to generate them are largely absent under physiological conditions. Moreover, the upper boundary of parasympathetic influence on heart rate is not sharply defined, and some degree of frequency-domain overlap between vagal modulation and HRF is likely. In healthy individuals, therefore, the rapid beat-to-beat fluctuations captured by HRF most plausibly reflect vagally mediated dynamics rather than a distinct, autonomically independent intrinsic rhythm.

Within this framework, the strong and consistent association between PIP and the 2UV% symbolic pattern warrants further consideration. The 2UV% pattern reflects alternating beat-to-beat changes and is typically interpreted as a marker of vagally mediated, high-frequency variability. From a signal-structure perspective, however, an increased prevalence of 2UV% also denotes a higher occurrence of rapid directional reversals in successive RR intervals, behavior conceptually aligned with a fragmented HRV. Under physiological conditions, therefore, 2UV% and HRF indices may represent overlapping manifestations of the same underlying respiratory-driven, vagally mediated dynamics. This interpretation also raises the possibility that 2UV% may carry a distinct physiological meaning in pathological contexts. In conditions characterized by structural or functional alterations of the sinoatrial node or atrial tissue (such as fibrosis, inflammation, or impaired intercellular coupling) an increased prevalence of alternating beat-to-beat patterns would more plausibly reflect intrinsic cardiac instability than heightened autonomic modulation (Stein et al., 2008; Costa et al., 2017a, b). Under these circumstances, a dissociation between 2UV% and established vagal indices would be expected, and elevated 2UV% could emerge as a marker of non-autonomic, intrinsic mechanisms driving heart rate fragmentation.

Accordingly, while the present findings support a predominantly autonomic, vagally mediated origin of both HRF and 2UV% under physiological conditions, they also raise the possibility that these indices may carry different mechanistic meanings in diseases. Previous studies employing complexity measures have shown that alterations in heart rate variability can occur independently of conventional autonomic markers. In particular, in the Multi-Ethnic Study of Atherosclerosis, fragmentation indices predicted incident atrial fibrillation independently of conventional risk factors, a finding attributed to disrupted sinoatrial pacemaker function rather than to autonomic modulation (Costa et al., 2021b). Consistently, in the PROOF-AF study, heart rate fragmentation assessed in an elderly general population improved the identification of individuals at risk of atrial fibrillation (Guichard et al., 2025). In both cohorts, the populations were older than our cohort or harbored structural and electrical substrates, conditions in which the intrinsic determinants of fragmentation, such as pacemaker cell dysfunction, fibrosis, and impaired intercellular coupling, are expected to predominate. Under these circumstances, elevated fragmentation and an increased prevalence of alternating beat-to-beat patterns would be expected to dissociate from vagally mediated indices, in contrast to the tight coupling observed in the present healthy cohort. Accordingly, HRF-derived metrics and 2UV% may capture aspects of cardiac rhythm organization that extend beyond autonomic modulation alone, consistent with the notion that disease does not simply reflect reduced variability but rather an altered structure of physiological dynamics, a hypothesis that warrants further investigation in pathological populations. Taken together, these observations reinforce a context-dependent interpretation, in which the rapid beat-to-beat dynamics captured by HRF and by the 2UV% pattern reflect predominantly vagal modulation under physiological conditions but may shift toward intrinsic sinoatrial instability as structural and electrical alterations accumulate, as well as the importance of not relying on a single method of HR analysis, but rather approaching interpretation through a holistic perspective that integrates multiple HRV and HRF indices.

Results from the controlled breathing protocol further addressed whether the so-called ultra-high-frequency components of HRF represent the upper boundary of vagally mediated HRV, rather than a genuinely distinct intrinsic rhythm. As a matter of fact, all variations corresponding to high frequencies (HF, 2UV%, PIP, and other indices related to more fragmented rhythms) decreased during this autonomic maneuver, in favor of an increase in low-frequency variations (LF, 0V%, 2LV%).

As anticipated, slow-paced breathing produced clear signs of enhanced vagal modulation, with increased overall HRV and a spectral shift of power from the high- to the low-frequency band. Notably, the resulting increase in the LF/HF ratio should not be interpreted as indicative of sympathetic dominance. During controlled breathing at 0.1 Hz, the respiratory-driven oscillations ordinarily confined to the high-frequency band are displaced into the low-frequency range by the slower breathing rate, an expected consequence of this protocol that diverges from the conventional interpretation of the LF/HF ratio (Eckberg, 2003).

The amplitude and phase of respiratory sinus arrhythmia are themselves frequency-dependent (Angelone et al., 1964; Saul et al., 1989); at the low breathing rate imposed here, the assumptions commonly applied at spontaneous respiratory frequencies no longer hold, which reinforces that the time- and spectral-domain indices observed during this protocol should not be read through the conventional framework. It is also worth noting that 0.1 Hz corresponds to the resonance frequency of the cardiac arm of the baroreflex, which is the principal mechanism amplifying the magnitude of heart period variability during slow-paced breathing (Baselli et al., 1994). This baroreflex engagement does not confound the present interpretation. The rapid, beat-to-beat oscillation it produces is effected through the vagal branch of the baroreflex arc, so that the modulation reaching the sinoatrial node remains autonomic in origin. The baroreflex thus specifies the route through which the vagally mediated oscillation is amplified at this frequency, but it does not alter the conclusion that the fragmentation indices track the spectral location of this vagally mediated oscillation, rather than an intrinsic sinoatrial rhythm.

Controlled breathing induced a concurrent increase in 2LV% and reduction in 2UV%, while the most fragmentation-sensitive HRF indices all decreased. As 2LV% captures oscillatory activity across an intermediate-to-high frequency range, it remained sensitive to the enhanced vagal modulation induced by slow-paced breathing. However, the concurrent reductions in 2UV% and PIP, as well as in other indices related to ultra-high-frequency variations (W_2_, W_3_, W^H^, and W^M^), reflect the shift of respiratory-driven dynamics toward slower frequencies imposed by the protocol, rather than a true attenuation of vagal cardiac tone. These observations are consistent with the interpretation proposed above and further illustrate how respiratory frequency can influence the expression of HRF indices in healthy individuals.

Taken together, these observations indicate that HRF, in healthy subjects, reflects the rapid, beat-to-beat component of vagal modulation of the sinoatrial node. This relationship, however, is conditional on the spectral location of the respiratory oscillation. Under physiological conditions, when respiration occupies the high-frequency band, the vagally mediated oscillation and the high-frequency content of the RR signal coincide, and HRF tracks conventional vagal markers, as observed during spontaneous breathing and graded tilt. Controlled breathing at 0.1 Hz dissociates these two features by displacing the respiratory oscillation into the low-frequency band. Under this condition, overall vagal modulation increases while HRF indices decrease, because the rapid oscillatory signature captured by HRF is relocated outside the band in which it is expressed. Rather than contradicting the vagal interpretation, this dissociation delimits it: HRF reflects vagal modulation specifically through its high-frequency oscillatory expression, and the 0.1 Hz maneuver reveals the boundary of this relationship by moving that expression to a lower frequency. The strong correlation between PIP and the 2UV% symbolic pattern, a recognized marker of vagally mediated modulation, further supports the vagal origin of the rapid dynamics probed by HRF under physiological conditions.

Recent population-based studies have reported associations between elevated HRF and adverse clinical outcomes, including kidney function decline and atrial fibrillation, findings that have been interpreted as consistent with impaired parasympathetic function and broader neuroautonomic dysregulation (Costa et al., 2025; Guichard et al., 2025), suggesting that HRF may not be entirely independent of autonomic regulation. The present findings extend these emerging observations by demonstrating that HRF is sensitive to autonomic adjustments, particularly shifts in vagal activity, even in healthy individuals. This underscores the importance of population context when interpreting HRF indices.

Because prior characterizations of HRF have been conducted predominantly in older or clinically affected populations, the present study establishes a physiological reference frame for HRF behavior in the absence of disease-related structural alterations. This reference frame, however, is necessarily bounded by the characteristics of the sample. The modest number of participants and their narrow age range (29 ± 4 years) mean that the present findings describe HRF at the physiological end of the spectrum, and their extrapolation to older individuals or to clinical populations, in whom age-related and disease-related sinoatrial alterations become prominent, requires dedicated investigation. Future research in clinical populations will be essential to determine the extent to which the HRF–autonomic interplay observed here is preserved or modified under pathological conditions.

## Limitations

Several limitations should be acknowledged. The exclusion of individuals with supraventricular or ventricular ectopy, although necessary to ensure stable sinus rhythm for the symbolic and fragmentation analyses, may have removed the very subjects in whom HRF would reflect intrinsic, non-autonomic dynamics, likely contributing to the predominantly vagal profile observed here. Menstrual cycle phase was not recorded in female participants and therefore could not be accounted for in the present analysis. In addition, the study included a relatively small sample of young healthy adults. Consequently, the present findings should be interpreted as characterizing HRF behavior under physiological conditions and may not be directly generalizable to older individuals or clinical populations, in whom HRF has been more extensively investigated.

The thoracic belt allowed reliable assessment of respiratory frequency but was not calibrated to provide quantitative measurements of tidal volume. Therefore, changes in tidal volume cannot be formally excluded.

Finally, although count-based and symbolic indices such as PIP and 2UV% are largely independent of the mean RR interval, unlike magnitude-based indices such as SDNN and RMSSD (Silva et al., 2017), the 5 ms threshold applied in the HRF analysis introduces a partial amplitude dependence. A contribution of the higher heart rate at greater tilt angles to the reduction in fragmentation indices therefore cannot be entirely excluded, although the parallel behavior of 2UV%, which is insensitive to mean heart rate, argues against a purely mechanical effect.

## Conclusion

This study provides the first physiological evidence that cardiac autonomic modulation, particularly parasympathetic activity, significantly influences HRF in healthy subjects. The consistent co-variation and correlation between HRF and vagally mediated HRV indices indicate that both metrics probe overlapping aspects of cardiac parasympathetic regulation. This influence is expressed through the high-frequency oscillatory content of the RR signal: under physiological conditions, when respiration happens in the high-frequency band, HRF and vagally mediated HRV indices co-vary and probe overlapping aspects of cardiac parasympathetic regulation. Controlled breathing at 0.1 Hz delimits this relationship by relocating the respiratory oscillation to the low-frequency band, revealing that HRF tracks vagal modulation specifically through its high-frequency expression. Furthermore, the elevated occurrence of the 2UV% pattern in symbolic dynamics analysis may reflect an underlying fragmented HRV structure and, under specific pathological circumstances, could also capture non-autonomic, intrinsic cardiac alterations. Future investigations are warranted to elucidate the mechanistic basis of these associations and to determine whether HRF can serve as a sensitive early indicator of the transition from physiological autonomic modulation to pathological cardiac dysregulation.

## Data Availability

The raw data supporting the conclusions of this article will be made available by the authors, without undue reservation.
